# Influence of late pruning practice on two red skin grapevine cultivars in a semi-desert climate

**DOI:** 10.3389/fpls.2023.1114696

**Published:** 2023-02-08

**Authors:** Corrado Perin, Pankaj Kumar Verma, Gil Harari, Yedidya Suued, Matanya Harel, Danielle Ferman-Mintz, Elyashiv Drori, Yishai Netzer, Aaron Fait

**Affiliations:** ^1^ Dipartimento di Agronomia Animali Alimenti Risorse Naturali e Ambiente, University of Padova, Padova, Italy; ^2^ Albert Katz International School for Desert Studies, Jacob Blaustein Institutes for Desert Research, Ben-Gurion University of the Negev, Midreshet Ben-Gurion, Israel; ^3^ Carmel Winery, Soham, Israel; ^4^ Eastern Regional R&D Center, Ariel, Israel; ^5^ Chemical engineering Department, Ariel University, Ariel, Israel; ^6^ Albert Katz Department of Dryland Biotechnologies, French Associates Institute for Agriculture and Biotechnology of Drylands, Jacob Blaustein Institutes for Desert Research, Ben-Gurion University of the Negev, Midreshet Ben-Gurion, Israel

**Keywords:** grape must, wine, UPLC-MS, late shoot pruning, secondary metabolites, anthocyanin, stilbene

## Abstract

Continually increasing global temperature could severely affect grape berry metabolite accumulation and ultimately wine polyphenol concentration and color intensity. To explore the effect of late shoot pruning on grape berry and wine metabolite composition, field trials were carried out on *Vitis vinifera* cv. Malbec and cv. Syrah grafted on 110 Richter rootstock. Fifty-one metabolites were detected and unequivocally annotated employing UPLC-MS based metabolite profiling. Integrating the data using hierarchical clustering showed a significant effect of late pruning treatments on must and wine metabolites. Syrah metabolite profiles were characterized by a general trend of higher metabolite content in the late shoot pruning treatments, while Malbec profiles did not show a consistent trend. In summary, late shoot pruning exerts a significant effect, though varietal specific, on must and wine quality-related metabolites, possibly related to enhanced photosynthetic efficiency, which should be taken into consideration when planning mitigating strategies in warm climates.

## 1 Introduction

Grape berry metabolite composition is crucial for producing premium-quality wine with regional characteristics, as the grape berry metabolic status is intimately associated with environmental and seasonal variation (Bokulich et al., 2016). Climate change forecasts predict significant thermal increases for Mediterranean climate zones, which will intensify the effects on vine phenology and berry composition. Elevated ambient temperature is one of the most prominent adverse environmental factors affecting wine quality (Gutiérrez-Gamboa et al., 2021). Elevated temperature causes the compression of the harvest period (Rienth et al., 2021), or varietal shifts in phenology of certain developmental phases (Gashu et al., 2020). These phenological changes can directly impact grape metabolism entailing significant composition changes (Abeysinghe et al., 2019; Venios et al., 2020), e.g., increased content of total soluble solids (TSS), increased pH, alteration in the amino acid profile as well as a negative effect on the accumulation of phenolic compounds (Kliewer, 1971; Kliewer, 1977; Baeza et al., 2019). Taken together, the impact on the wine quality and regional characteristics can be severe (Rienth et al., 2016), thus, to produce grapes with the desired metabolic balance and mitigate the climate challenges, optimizing proper viticulture practices will be fundamental.

A possibility for adapting viticulture to impending climate changes is to improve photosynthetic efficiency during grape ripening processes, e.g., by postponing winter pruning from dormancy to post-dormancy. Other field practices capable of modifying vine phenology include post-veraison leaf removal, post-bud-break pruning, and severe trimming or forcing vine regrowth (Allegro et al., 2019; Buesa et al., 2019). Late shoot pruning, also referred to as delayed winter pruning, is a pruning technique practised in the spring after the bud break occurs to tune growth and yield. Recently, its application has been proposed as a significant tool for optimizing vine growth in warm regions, containing crop yield, and improving wine quality (Netzer et al., 2022). It was shown that late shoot pruning was also associated with improved photosynthetic efficiency and lower crop load. However, the effect of the practice seems not to be consistent between varieties, e.g. increased yield of Merlot in New Zealand (Friend and Trought, 2007), while the decreased yield of cv. Sangiovese in Italy (Frioni et al., 2016). More importantly, slight or no information exists from different varieties on the response of berry and wine chemistry following late pruning treatments from within the same experimental setup.

The goal of this work was to investigate the effect of incremental late shoot pruning on the chemical composition of must and wine from Syrah and Malbec varieties. Earlier works showed phenological syncing between the treatments, but lower yield and higher quality in the late shoot pruned vines (Netzer et al., 2022). Using a liquid chromatography-mass spectrometry-based approach we compared the varietal profiles of must and wine from late shoot pruning (LSP1, LSP2, LSP3), standard winter pruning (WP), and winter pruning and cluster thinning (WP+T) pruning treatments.

## 2 Materials and methods

### 2.1 Experimental site and plant material

The study was performed in 2017 and 2018 on an experimental vineyard planted in 2010 in Ayalon Valley, Israel (31°86’N; 35°01’E), 186 m above sea level. The experimental layout was a randomized complete block design with five treatments, and it was replicated four times. Row orientation was east/west, with a slight tendency to the south and vine and row spacing 1.5 and 3 m, respectively (4.5 m^2^/vine) (**Figure S1**). Pest management, irrigation and fertilization in the vineyard were applied according to standard local agricultural practices. Must and wine samples from the 2017 and 2018 seasons were collected and analyzed by LC-MS.

### 2.2 Winter pruning and late shoot pruning

The winter pruning and late shoot pruning treatments were performed exactly as described by (Netzer et al., 2022) on the vines of *Vitis vinifera* cv. Syrah, and *Vitis vinifera* cv. Malbec was grafted on 110 Richter rootstock. Briefly, five pruning treatments including LSP1 (pruning after 1 week of bud break), LSP2 (pruning after 2 weeks of bud break), LSP3 (pruning after 3 weeks of bud break), WP+T (standard winter pruning and cluster thinning as control), and WP (standard winter pruning as control). These pruning treatments were randomly assigned to blocks, so there were 11*4 vines for each treatment and treatments were applied to the same vines in both years.

### 2.3 Berry sampling and metabolite analysis in must and wine

Each year, berries from all the treatments were harvested at a more or less, similar TSS (°Brix) value. Must samples were collected from bulk unfermented juice after settling for an hour and then decanting the liquid. The wine samples were obtained from the micro vinification process of the corresponding treatments (Drori et al., 2017). Must and wine from both the 2017 and 2018 seasons were then analyzed by LC-MS as described below.

#### 2.3.1 Metabolite extraction

For the metabolite analysis, must samples were pulverized by mortar and pestle while keeping the material frozen by pouring liquid nitrogen. Approximately 200 mg of must powder was weighed and lyophilized in ScanVac CoolSafe™ (Labogene, Denmark, https://www.labogene.com). While wine samples were directly lyophilized in polypropylene tubes without pulverization. Metabolites were then extracted by adding 1ml pre-chilled methanol: chloroform: water extraction solution (2.5:1:1 v/v), following the protocol described by (Hochberg et al., 2013; Degu et al., 2014). After that, internal standards (300μl of 1mg/ml ampicillin in water and 380μl of 1mg/ml corticosterone in methanol) were subsequently added as described (Degu et al., 2016). The mixture was then briefly vortexed, 100μl of methanol was added and then placed on a horizontal shaker for 10min at 1000rpm. The samples were sonicated for 10min in an Elmasonic S30 ultrasonicator (Elma Singen, Germany, http://www.elma-ultrasonic.com/) and centrifuged at 14000rpm for 10 min (Centrifuge 5417R, Eppendorf SE, Hamburg, Germany, https://www.eppendorf.com). The supernatant was then decanted into new tubes, mixed with 300μl of chloroform and 300μl of MiliQ water (Millipore, MA, USA, https://www.merckmillipore.com), vortexed for 10s and then centrifuged again for 5min at 14000rpm. The water/methanol phase, obtained from the extraction protocol (around 1ml), was collected, and filtered using 0.22 μm (Millipore, MA, USA, https://www.merckmillipore.com) and stored in vials for UPLC analysis.

#### 2.3.2 UPLC analysis

Each sample was analyzed twice in an Ultra Performance Liquid Chromatography coupled with a Quadrupole Time-of-Flight Mass-Spectrometer (UPLC-QTOF MS, Waters, MA, USA, https://www.waters.com/) system operating in both positive and negative ion modes, alternatively (**Table S6**). The MassLynx™ software (Waters, MA, USA, https://www.waters.com/) version 4.1 was used for UPLC system control and data acquisition. The acquired raw data were processed using the MarkerLynx application manager (Waters, MA, USA, https://www.waters.com/) as described by Hochberg et al., 2013. Metabolite’s annotation was based on the mass fragment (mass/charge; m/z), their retention time (RT), and the comparison with the internal library as well as the current scientific literature (Degu et al., 2014). In addition, metabolites were also annotated based on fragmentation patterns crossed with the ChemSpider metabolite database (www.chemspider.com).

#### 2.3.3 Data normalization and statistical analysis

The chemical feature’s peak area detected by the instrument was normalized by internal reference for UPLC analysis (i.e., ampicillin and corticosterone in the negative and positive ion mode, respectively). We used two different internal standard references as analytes have different ionizing efficiency in positive and negative ion modes, e.g., some analytes are not ionized in negative mode, but very well ionized in positive mode. We annotated metabolites uniquely in both negative and positive ion modes. We used corticosterone as an internal standard reference to normalize data of metabolites annotated in positive ion mode, and due to poor ionization of corticosterone in negative mode, we use another internal standard ampicillin as a reference to normalize data of metabolites annotated in negative mode to minimizes the variability in sample preparation as well as variability generated by the instrument such as injection volume. Further accuracy was acquired by normalizing samples’ values to the relative dry weight (post-lyophilization). Therefore, data refer to the relative metabolite abundance based on ion counts. For hierarchical clustering representation, mean values were used, and metabolite values were ln (x+1) transformed. In addition, unit variant scaling was applied for this analysis.

The two datasets (wine and must) were utilized for statistical analysis based on i) hierarchical clustering as a multivariate approach to the study; ii) analysis of variance for each detected metabolite (multifactorial ANOVA) considering the effect of three factors (pruning, cultivar, and year), the effect of their interactions (pruning × cultivar, pruning × year, cultivar × year, pruning × cultivar × year); iii) Pearson correlation analysis for investigating on the putative relationships among metabolites both as must and wine autocorrelations and must-to-wine bipartite correlation. Statistical analysis and data visualizations were performed in the R environment (R Core Team, 2022a) using R studio IDE (R Studio Team, 2022b).

## 3 Results

### 3.1 Pruning exerts a significant effect on must and wine metabolite status

Standard winter pruning was conducted according to common agricultural practice in mid-February. In examining the seasonal patterns of phenological development throughout the three seasons, previous findings showed that late pruning treatments re-started the phenological process at a similar pace (Netzer et al., 2022). A one-week difference between dates of late pruning, conducted per the relevant treatments, was evident in the phenological pattern until mid-May. Nonetheless, a significant increase in the pace of development in the later winter pruning treatments was apparent from mid-May until the end of June, bringing all treatments to sync at veraison (stage 35).

**Figure 1:**
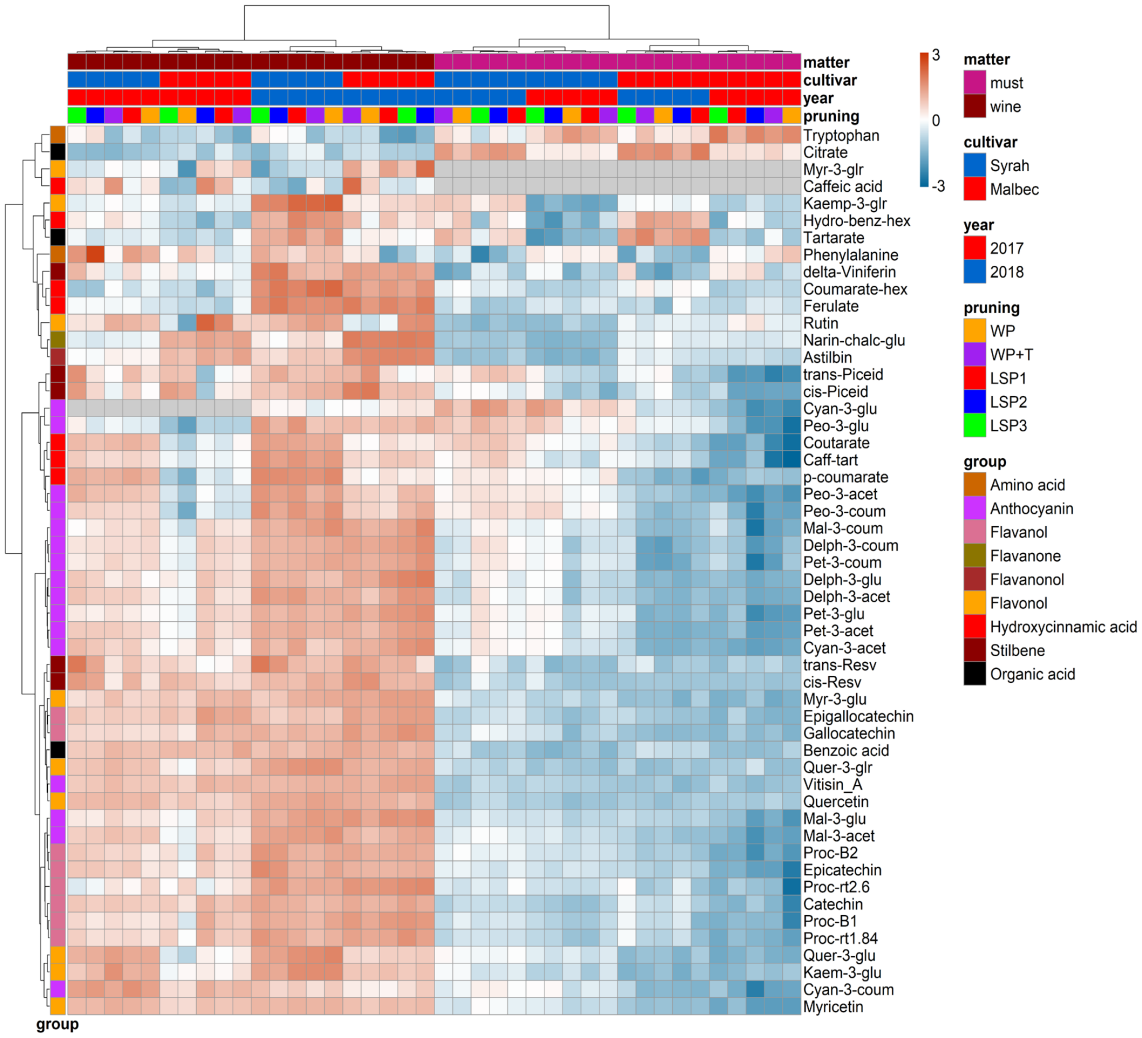
Hierarchical clustering heatmap obtained from cluster analysis. Cluster sharply divided the samples based on the kind of sample (wine vs must) then *cultivar*, year and pruning treatments. Samples from the most extreme late shoot pruning treatments (“LSP3” and “LSP2”) and samples from the no-late pruning treatments (“WP+T” and “WP”) seemed to form two different groups. The late shoot pruning treatment “LSP1” alternatively clustered with one of the two groups.

**Figure 2:**
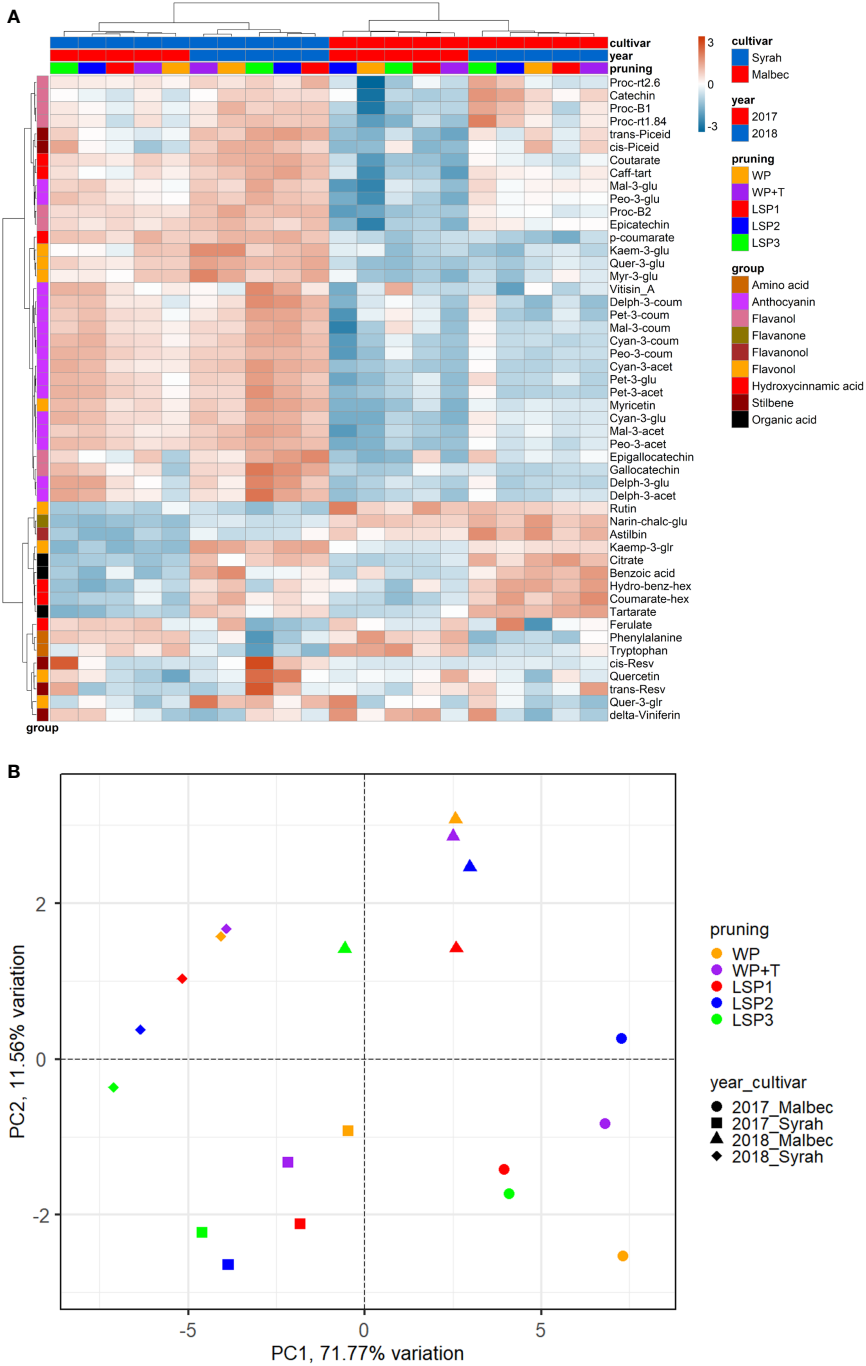
Heatmap and 2D PCA score plot of annotated LC-MS based metabolites in must Samples. **(A)** Hierarchical clustering heatmap obtained from the cluster analysis sharply divided the samples into cultivar and year and pruning. **(B)** PCA score plot from metabolite data including *cultivar* and year and pruning treatments. Samples from the most extreme late shoot pruning treatments (“LSP3” and “LSP2”) and samples from the no-late pruning treatments (“WP+T” and “WP”) seemed to form two different groups. The late shoot pruning treatment “LSP2” alternatively clustered with one of the two groups.

**Figure 3:**
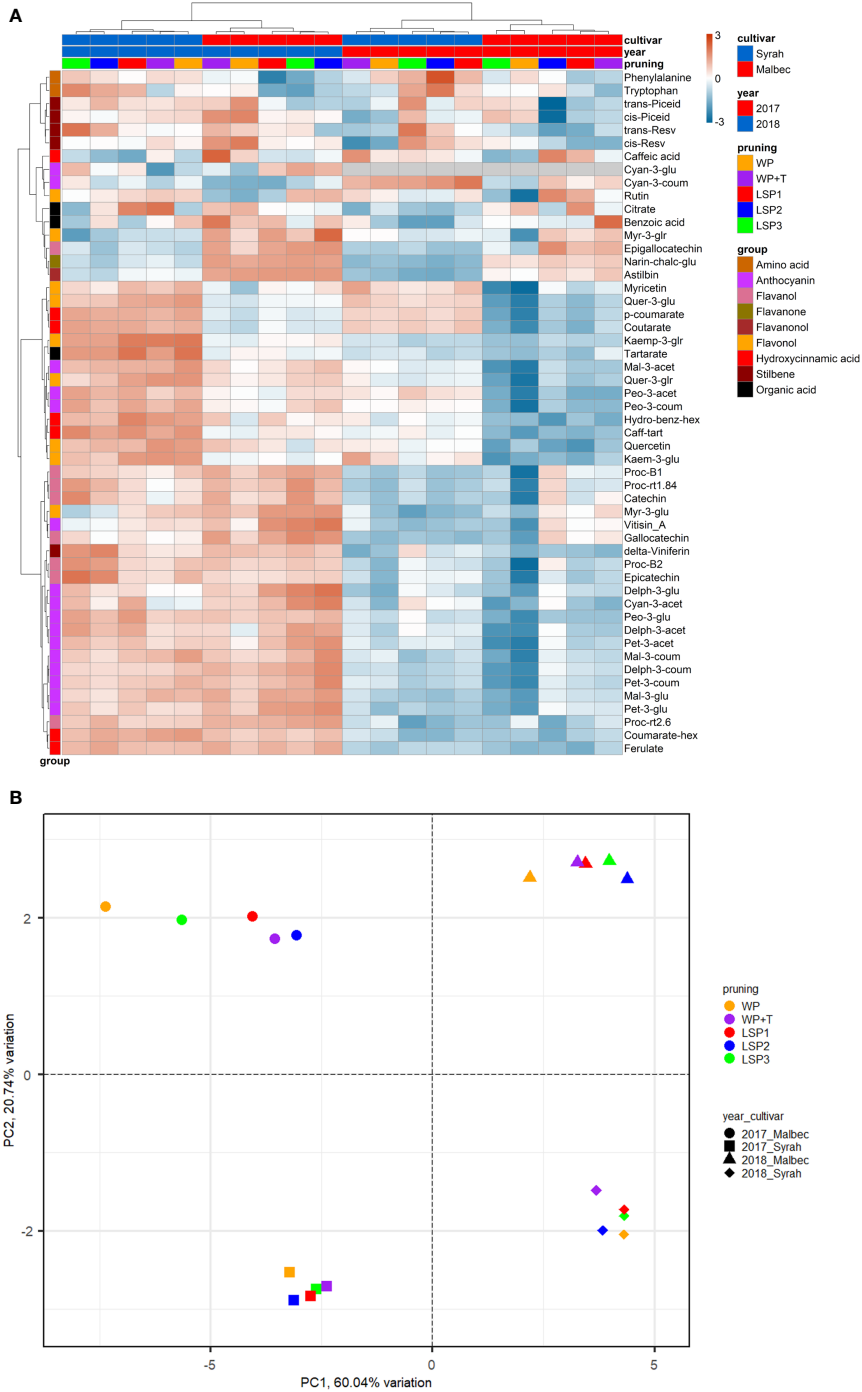
Heatmap and 2D PCA score plot of annotated LC-MS-based metabolites in wine Samples. **(A)** Hierarchical clustering heatmap obtained from the cluster analysis sharply divided the samples by year and then cultivar and pruning. **(B)** PCA analysis suggests that the wine samples were clustered by ‘year’ (2017 *vs* 2018) and then by ‘cultivar’ (Malbec vs Syrah).

To test if the phenological syncing was reflected in the must and wine chemistry, we analysed samples of must and wine using LC-MS-based protocol across the entire experimental setup, i.e., a total of 160 samples. Fifty metabolites were consistently identified and unequivocally annotated. Metabolites primarily belong to polyphenols, including anthocyanins, flavonoids, phenolic acids, and stilbenes (**Figure 1**; **Tables S1**, **S2**). Hierarchical clustering was performed using the metabolic data of must and wine. The dendrogram obtained after the cluster analysis sharply divided the samples based on their origin (wine vs must) (**Figure 1**). Next, the samples were clustered by ‘cultivar’ (Malbec vs Syrah), and then by ‘year’ (2017 vs 2018). The opposite pattern was shown for the wine samples, i.e., first by ‘year’, then by ‘cultivar’. Within each sub-cluster (pruning-cultivar-year combination), samples from the most extreme late shoot pruning treatments (LSP3 and LSP2) and samples from the no-late pruning treatments (WP+T and WP) formed two separate groups (**Figure 1**). When repeating the analysis for must and wine samples separately, clustering was observed primarily by ‘cultivar’ (Malbec vs Syrah), then by year in the must (**Figures 2A, B**). In the wine, clustering was observed primarily by treatment (**Figures 3A, B**). In both datasets, five main groups of metabolites were identified based on their pattern of change i) amino acids, (ii) anthocyanins (iii) flavonoids (flavanols, flavanones, flavanonol and flavonols) (iv) hydroxycinnamic acids and (iv) stilbenes. The analysis of variance revealed that different pruning treatments had a significant effect on most of the detected metabolites (**Tables S1**, **S2**), but all metabolic groups were differentially affected, with the most influenced metabolic classes being anthocyanins (**Figures S2A–D**) and stilbenes (**Figures S3A–D**). The highest relative content of anthocyanins and stilbenes were detected in the most extreme late pruning treatment in must, i.e., vines pruned three weeks after bud-break (LSP3). For instance, a general trend in Syrah must sample was observed from the “late shoot pruning” treatments (i.e., LSP3, LSP2, and LSP1) which measured higher anthocyanin and stilbene content in comparison with the standard pruning treatments, i.e., WP+T and WP. Among the anthocyanins, Pet-3-glu, Cyan-3-glu, Peo-3-glu, Delph-3-acet, Pet-3-acet, Cyan-3-acet, Peo-3-acet, Peo-3-coum showed higher content in the LSP3 Syrah must (**Figure S2A**), while Delph-3-glu, Delph-3-acet, Peo-3-acet, accumulated in LSP3 wine samples (**Figure S2B**). On the other hand, in Malbec, the LSP3 late-pruning treatments reported higher anthocyanin abundance as compared with the “no-late-pruning” with higher accumulation in LSP3 in must and LSP2 treatment in wine samples, but it significantly depends on the season (**Figures S2C, D**). Among the stilbenes trans-piceid, cis-piceid, trans-resv, cis-resv and delta-viniferin showed higher content in Syrah must and wine samples which were also affected by seasonal variations (**Figures S3A, B**), on the other hand, Malbec showed higher content of trans-piceid and cis-piceid in both must and wine in 2017’s most extreme late pruning treatment, i.e., LSP3 (**Figures S3C, D**). Flavanols such as Proc-B2 and epicatechin followed the same trend as anthocyanins in the Syrah wine sample (**Figure 3A**). Flavanols like Proc-B1, Proc-rt1.84, Proc-B2, catechin, epicatechin, epigallocatechin and gallocatechin followed a general trend with the highest accumulation in LSP3 2018 in Malbec must (**Figure 2A**) and Proc-rt1.84, epigallocatechin in wine samples (**Figure 3A**). Among flavonols, quercetin and myricetin were higher in the late pruning treatments, in Syrah must samples (**Figure 2A**). Notably, naringenin-chalcone and astilbin were not affected by the pruning treatments. The statistical analysis using *post hoc* Tukey’s test revealed a significant difference between cultivars (Malbec vs Syrah) and between vintages (2017 vs 2018) (**Tables S1**, **S2**).

### 3.2 Factor interaction affected metabolite accumulation

Cultivar × treatment interaction mainly concerned the group of anthocyanins and stilbenes (**Tables S1**, **S2**). A general trend of higher metabolite content in the late pruning treatments was evident in must samples. All other metabolites either did not follow a common trend or were not significantly affected. Must flavanols, flavonols, and hydroxycinnamic acids were affected by cultivar and year interaction, while wine anthocyanins and flavonols were affected by cultivar and year interaction (**Table S1**). No effect from the interaction between pruning and year factors was detected except for the flavanol (Proc-rt1.84), the flavonol (myricetin-3-glucoside), and stilbene (cis-piceid). The triple interaction effect (treatment × cultivar × year) denotes a high sensitivity of secondary metabolites to external conditions. The triple interaction effect involved some classes of flavanols (Proc-B1, Proc-rt1.84, Proc-rt2.6, epigallocatechin) and hydroxycinnamic acids (p-coumarate, coutarate, ferulate, caff-tart), stilbene (trans-piceid, trans-resv) as well as flavanonol (astilbin) and the flavonol (myr-3-glu) in the must (**Figure 2**; **Table S1**). While in wine samples, flavanols (Proc-B1, Proc-rt1.84, Proc-B2) flavonols (quer-3-glu), and stilbene (delta-viniferin) were affected by the interaction between (treatment × cultivar × year) (**Figure 3**; **Table S2**). When repeating the ANOVA separating the two cultivars, Syrah reported more significant and consistent metabolite changes than Malbec in the anthocyanin and flavonol classes, whereas Malbec showed more significant variations in the hydroxycinnamic acids (**Figures 2**, **3**; **Table S2**). The number of metabolites affected by the cultivar-to-pruning interaction (cultivar × pruning) was higher in wine than in must. Besides stilbenes, cultivar-to-pruning interactions also concerned flavanols, flavonols, and more anthocyanins than in the must samples. Other interactions also regarded the combined effect of treatment and cultivar with vintage; they were generally noticed for some metabolites from every class except for hydroxycinnamic acids and flavanols.

### 3.3 Correlation analysis showed a positive correlation between anthocyanin and flavanols

**Figure 4:**
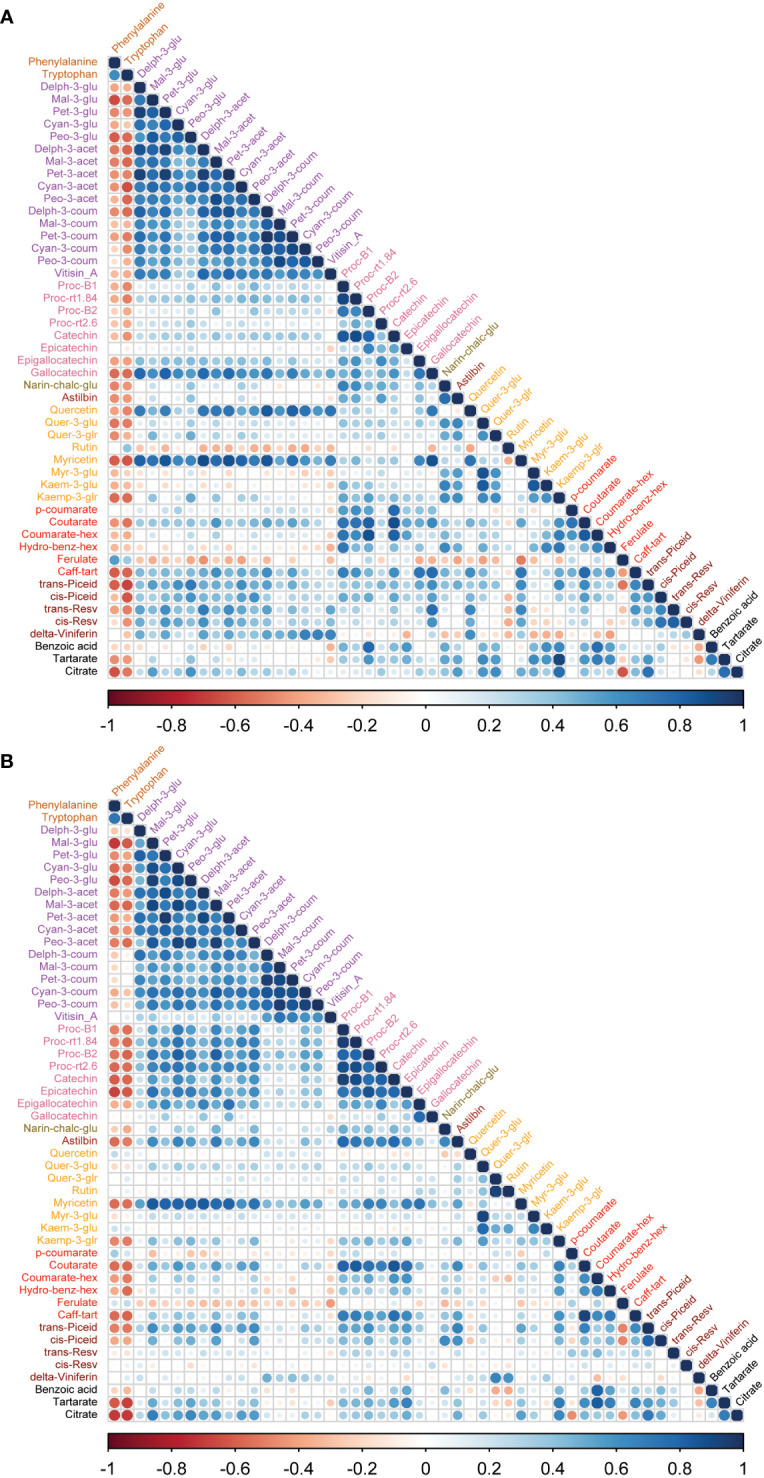
Correlation analyses between metabolites of two cultivars **(A)** Syrah must, most of the metabolites were positively correlated except rutin, myr-3-glu, kaem-3-glu, hydro-benz-hex, ferulate and benzoic acid which were negatively correlated. **(B)** Malbec must, most of the metabolites were positively correlated except p-coumarate, ferulate and cis-resv were negatively correlated with other metabolites. Amino acids were negatively correlated with most of the metabolites except rutin, ferulate in Syrah must, while quercetin, kaem-3-glu, p-coumarate and ferulate showed a positive correlation with amino acids. Vitisin_A was negatively correlated with most other metabolite groups.

**Figure 5:**
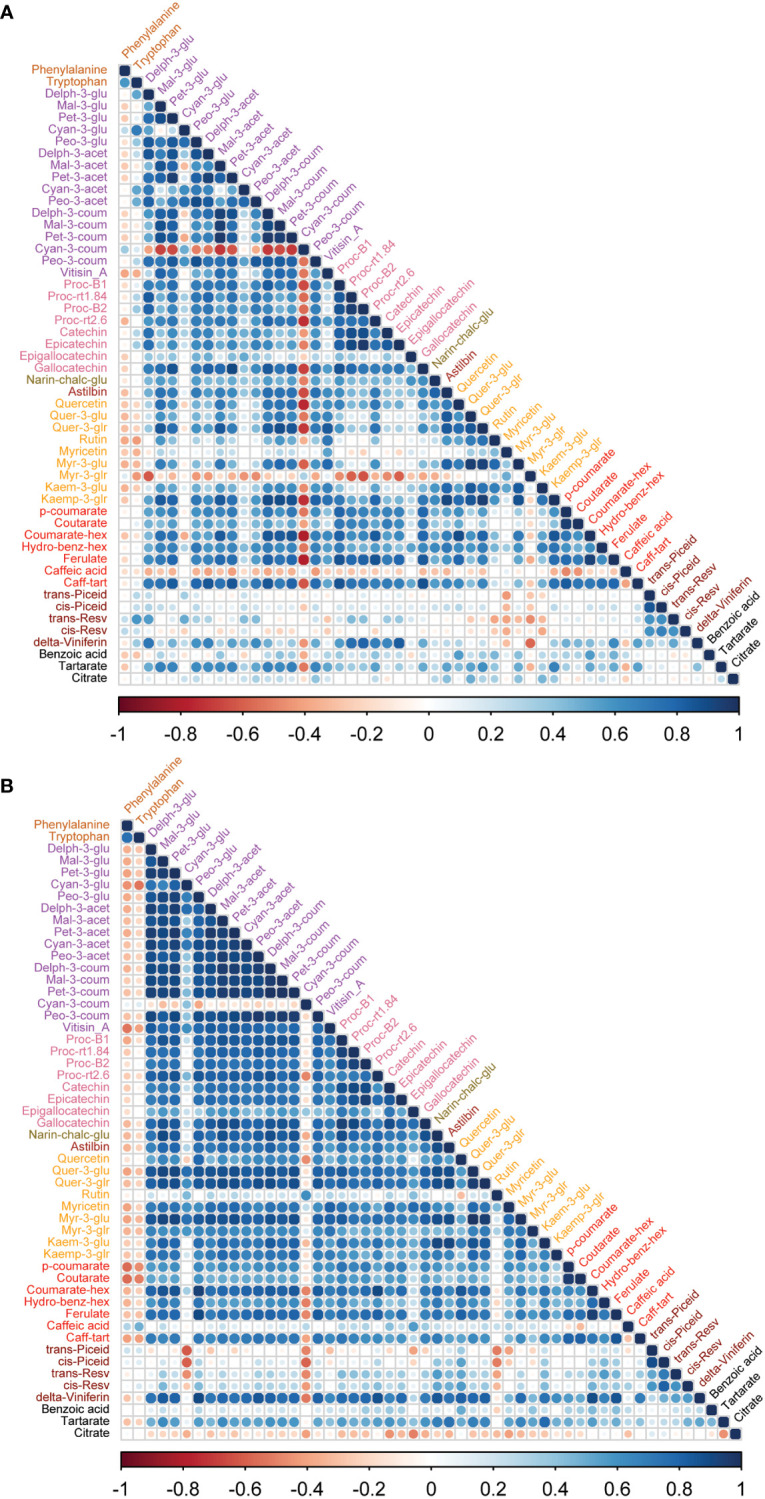
Correlation analyses between metabolites of wine samples from two cultivars. **(A)** In Syrah wine, most of the metabolites were positively correlated except cyan-3-coum, myr-3-glr and caffeic acid, which were negatively correlated. **(B)** In Malbec wine, most of the metabolites were positively correlated except cyan-3-coum, and citrate which were negatively correlated while rutin, caffeic acid, trans-piceid, cis-piceid, and benzoic acid had a weak correlation with other metabolites. Amino acids, phenylalanine and tryptophan were negatively correlated with most of the metabolites except trans-resv, cis-resv in Syrah wine, while caffeic acid, trans-piceid, and cis-piceid in Malbec wine showed a positive correlation with amino acids.

**Figure 6:**
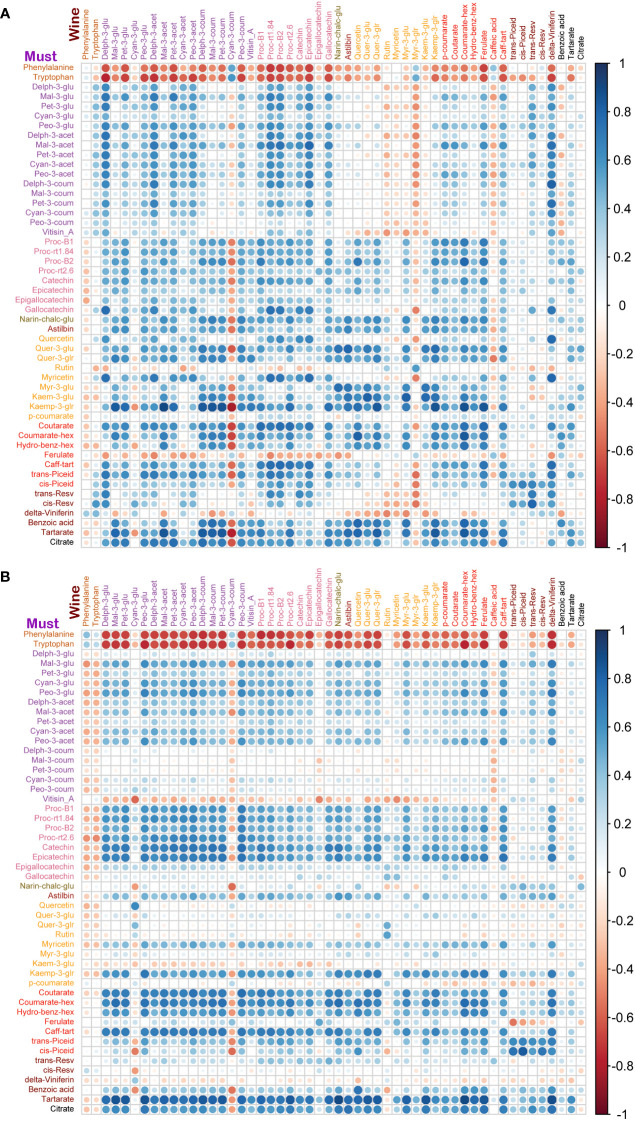
Correlation analyses between metabolites of must and wine samples from two cultivars. **(A)** In Syrah must and wine samples, must amino acids were negatively correlated with wine metabolites except for phenylalanine, cyan-3-coum, myr-3-glr and caffeic acid which were negatively correlated with most of the must metabolites. Must delta-viniferin was negatively correlated with most of the wine metabolites. **(B)** In Malbec must and wine samples, must amino acids were negatively correlated with wine metabolites except for amino acids and cyan-3-coum which were negatively correlated with most of the must metabolites. Must vitisin_A, kaem-3-glu and delta-viniferin were negatively correlated with most of the wine metabolites.

To study the coordination of metabolic processes concerning the pruning treatments, a correlation analysis using Pearson correlation was performed. The correlation analysis of must samples revealed high positive correlations among anthocyanins and flavanols. Vitisin_A showed a negative correlation with most of the metabolite classes except anthocyanins and stilbenes in must samples of Syrah and Malbec. Cultivar differences included a high number of positive correlations of gallocatechin and quercetin with anthocyanins in Syrah (**Figure 4A**; **Table S3** *Sheet1*), and strong positive correlations among flavanols in Malbec (**Figure 4B**; **Table S3** *Sheet2*) as well as strong positive correlations of astilbin, coutarate, caff-tart, and trans-piceid with the group of flavanol. Similar relations were also observed in the wine samples. When comparing the two cultivars separately, the Malbec correlation matrix reported higher indices in the number of correlations and their strength (**Figures 5A, B**; **Table S4** *Sheet1* and *Sheet2*). On the contrary, must and wine anthocyanins did not correlate well except for the wine delph-3-glu, delph-3-acet and peo-3-acet with all the must anthocyanins (**Figure 6**; **Table S5** *Sheet1* and *Sheet2*).

## 4 Discussion

The wine quality is reflected by the amount and composition of a large number of primary and secondary metabolites that shape its sensorial experience. Considering this view, it is likely that optimizing the environmental conditions during the grape ripening period will be required to produce good quality wine. These conditions can be achieved by modifying the viticulture practices in which late shoot pruning is the most important and economical way. Late shoot pruning can delay the phenology towards a cooler environment, thus able to minimize the effect of elevated temperature. The current study aimed to dissect the effect of late shoot pruning on differential metabolite accumulation in two red grapevine varieties, Malbec and Syrah.

In the LC-MS based metabolite profiles, amino acids did not follow a general trend, but their concentration decreased in late pruning, likely because these aromatic amino acids are precursors to the biosynthesis of secondary metabolites (Wang et al., 2017), and play a role in fruit and wine chemical quality. Among secondary metabolites, anthocyanins, flavanols, flavonols and stilbenes were the most affected groups by pruning treatments. Our findings suggest that flavanol accumulation is linked to the time of pruning in addition to environmental factors reported, e.g., light (Cortell and Kennedy, 2006; Reshef et al., 2017), soil conditions (Perin et al., 2020) temperature (Del-Castillo-Alonso et al., 2016b), UV-B radiation (Del-Castillo-Alonso et al., 2016a), and biotic stresses (Kuhn et al., 2013).

Among stilbenes, our study showed that resveratrol and viniferin were significantly modulated by the pruning treatments, with Syrah showing higher resveratrol content and Malbec in viniferin content. These results are consistent with previous studies showing the high plasticity of these phytoalexins (Anesi et al., 2015) and their relation to climate conditions (Dal Santo et al., 2016; Guerrero et al., 2020).

Our study shows a clear varietal specificity with respect to the effect of pruning treatments on the metabolite profile. For example, the role of the genotype is evident in anthocyanin’s pattern of change across the experimental setup, which follows a general trend of accumulation towards the later pruning treatments in Syrah. These results are supported by the findings that anthocyanin content changes in relation to climate conditions (Shah et al., 2021), and the effect of pruning on their accumulation (Zheng et al., 2017). However, many anthocyanins in Malbec did not follow a similar trend, suggesting that different varieties have specific mechanisms regulating anthocyanin metabolism.

The effectiveness of the time-dependent late pruning technique for improving grape composition was confirmed in both Syrah and Malbec. Grapes from the late shoot pruning treatments had a higher concentration of polyphenols in the must, which is in line with earlier observations of late pruning to decouple the ripening dynamics of sugars and phenolic substances (Sadras and Moran, 2012; Frioni et al., 2016; Silvestroni et al., 2018; Moran et al., 2021).

Wine metabolite profiling also confirmed the ameliorating effect of pruning, which is consistent with previous findings (Moran et al., 2018; Moran et al., 2019). Statistical analysis on a dataset of 51 detected metabolites revealed significant differences for all the metabolite classes except hydroxycinnamic acids. Differences between treatments were augmented in Syrah and slightly attenuated in Malbec wine, but in both cultivars, the higher color intensity and phenolic substances found in response to late shoot pruning are desirable attributes, as described also by Moran et al. (2019) for Syrah wine. Notably, in wine, the extreme late shoot pruning treatment (three weeks after bud break) was not characterized by the highest values of anthocyanin content as reported for the must samples. This suggests the existence of a non-linear relation between wine and must metabolites mediated by practices in the field. The year of production (vintage) and cultivar were major factors in the separation of wine samples on a PCA, while the treatment factor was more apparent in Malbec than in Syrah, which is not consistent with the must data. When considering the interaction between cultivar and treatments, Syrah positively modulates its metabolites in response to pruning treatments compared to Malbec. The results of this study also suggest a strong effect of late shoot pruning on both must and wine, consistent throughout the years.

Late shoot pruning can be effective when environmental conditions may limit the achievement of the desired grape ripeness, which is supported by earlier studies performed by (Mori et al., 2007; Sadras and Moran, 2012) as well as observations by Moran et al. (2018). Moran et al. (2018) found an interaction between the timing of pruning and temperature, whereby late pruning enhanced grape phenolic substances-to-sugars ratio in high-temperature vines but not in unheated control vines. Thus, the present study suggests that late shoot pruning by forcing the plant (1) to produce leaves later in the season, which will be considerably more efficient for photosynthesis and (2) having less sink load, improves carbon utilization by secondary metabolite biosynthetic pathways, including increased synthesis of phenolic substances.

## 5 Conclusion

The late shoot pruning shifted grapevine phenology and perturbed its metabolism. Nonetheless, a combination of a highly regulated berry phenological syncing, renewed leaf development with improved carbon assimilation capacity and a lower cluster load, led to improved metabolic features such as more stilbenes and flavonoids. Having that said, varietal-specific qualitative and quantitative metabolic alterations of the berry metabolism should be considered, and similar studies should be conducted before upscaling conclusions.

## Data availability statement

The original contributions presented in the study are included in the article/**Supplementary Material**. Further inquiries can be directed to the corresponding authors.

## Author contributions

CP: Conceptualization, Data curation, Methodology, Investigation. PV: Data curation, Methodology, Investigation, Writing – original draft, Writing – review & editing. GH: Formal analysis, Methodology, Investigation. YS: Formal analysis, Methodology, Investigation. MH: Formal analysis, Methodology, Investigation. DF-M: Formal analysis, Methodology, Investigation. ED: Conceptualization, Investigation, Methodology, Resources. YN: Conceptualization, Investigation, Methodology, Supervision, Resources, Project administration, Writing – original draft, Writing – review & editing. AF: Conceptualization, Investigation, Methodology, Supervision, Resources, Project administration, Writing – original draft, Writing – review & editing. All authors contributed to the article and approved the submitted version.

## Glossary

LSP3: Late shoot pruning 3 (three weeks after bud-break)
LSP2: Late shoot pruning 2 (two weeks after bud-break)
LSP1: Late shoot pruning 1 (one week after bud-break)
WP+T: Standard winter pruning and cluster thinning
WP: Standard winter pruning
Delph-3-glu: Delphindin-3-O-glucoside
Mal-3-glu: Malvidin-3-O-glucoside
Pet-3-glu: Petunidin-3-O-glucoside
Cyan-3-glu: Cyanidin-3-O-glucoside
Peo-3-glu: Peonidin-3-O-glucoside
Delph-3-acet: Delphinidin-3-O-(6′′-acetylglucoside)
Mal-3-acet: Malvindin-3-O-(6′′-acetylglucoside)
Pet-3-acet: Petundin-3-O-(6′′-acetyl-glucoside)
Cyan-3-acet: Cyanidin-3-O-(6′′-acetyl-glucoside)
Peo-3-acet: Peonidin-3-O-(6′′-acetyl-glucoside)
Delph-3-coum: Delphinidin-3-O-(6′′-p-coumaroyl-glucoside)
Mal-3-coum: Malvidin-3-O-(6′′-p-coumaroyl-glucoside)
Pet-3-coum: Petunidin-3-O-(6′′-p-coumaroylglucoside)
Cyan-3-coum: Cyanidin-3-O-(6′ ′-p-coumaroyl-glucoside)
Peo-3-coum: Peonidin-3-O-(6′′-p-coumaroyl-glucoside)
Proc-B1: Procyanidin B1
Proc-rt1.84: unknown (similar to Procyanidin retention time 1.84 minute)
Proc-B2: Procyanidin B2
Proc-rt2.6: unknown (similar to Procyanidin retention time 2.6 minute)
Narin-chalc-glu: Naringenin-chalcone-4-Oglucoside
Quer-3-glu: Quercetin-3-O-glucoside
Quer-3-glr: Quercetin-3-O-glucuronide
Rutin: Quercetin-3-O-rutinoside
Myr- 3-glu: Myricetin-3-O-glucoside
Myr-3-glr: Myricetin-3-O-glucuronide
Kaem-3-glu: Kaempferol-3-O-glucoside
Kaemp-3-glr: Kaempferol-3-O-glucuronide
Coumarate-hex: Coumarate-hexoside (p-Coumaric acid glucoside)
Hydro-benz-hex: Hydroxybenzoate hexoside (Hydroxybenzoic acid 4-O-glucoside)
Caff-tart: caffeoyl-tartaric acid
Resv: Resveratrol
