# Supplementary figures and images for: Influence of late pruning practice on two red skin grapevine cultivars in a semi-desert climate
Source: Front Plant Sci. 2023 Feb 8;14:1114696. doi: 10.3389/fpls.2023.1114696 (PMC9945113; doi:10.3389/fpls.2023.1114696)

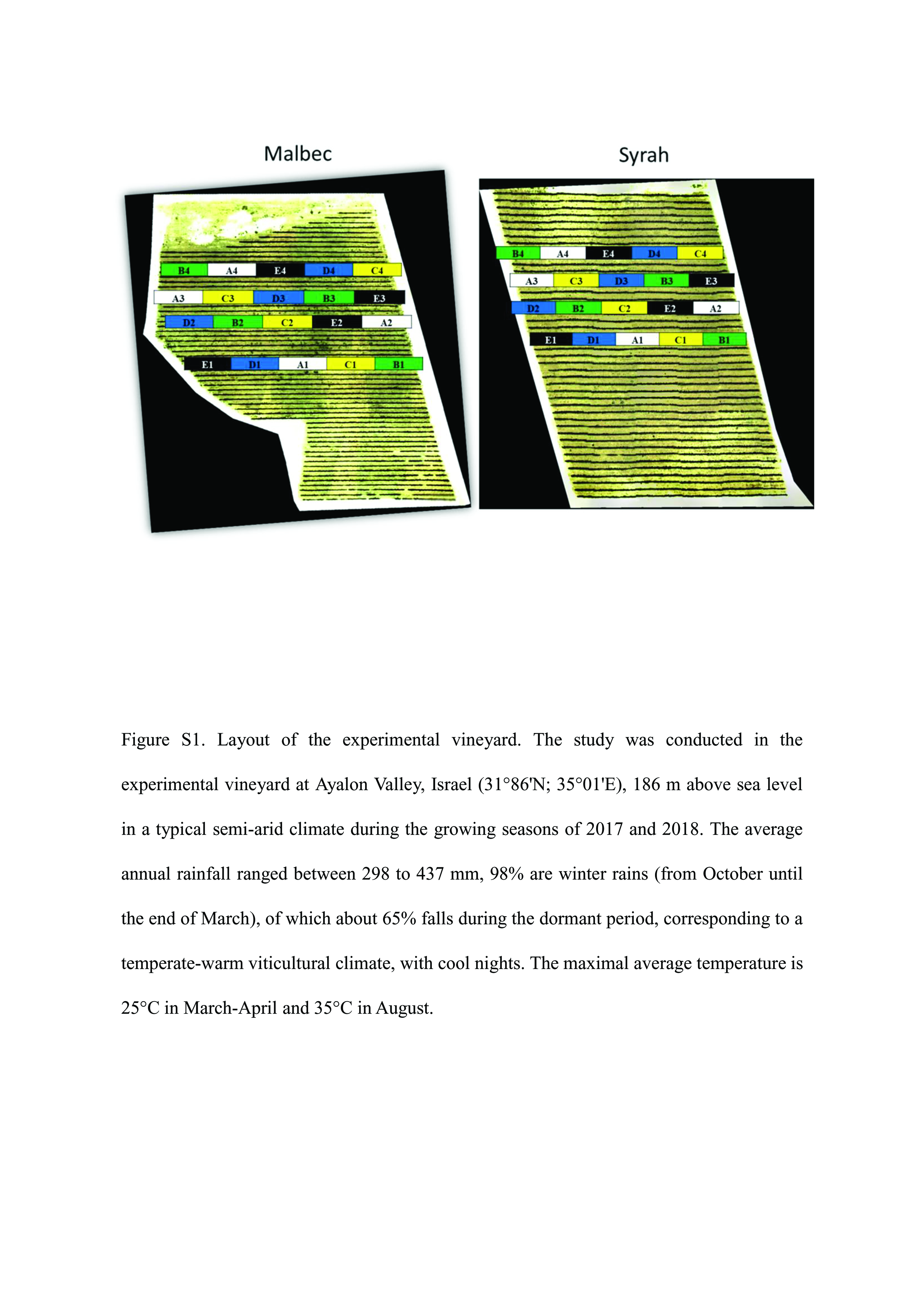

Supplement: Supplementary file 1 [file Image_1.tif]

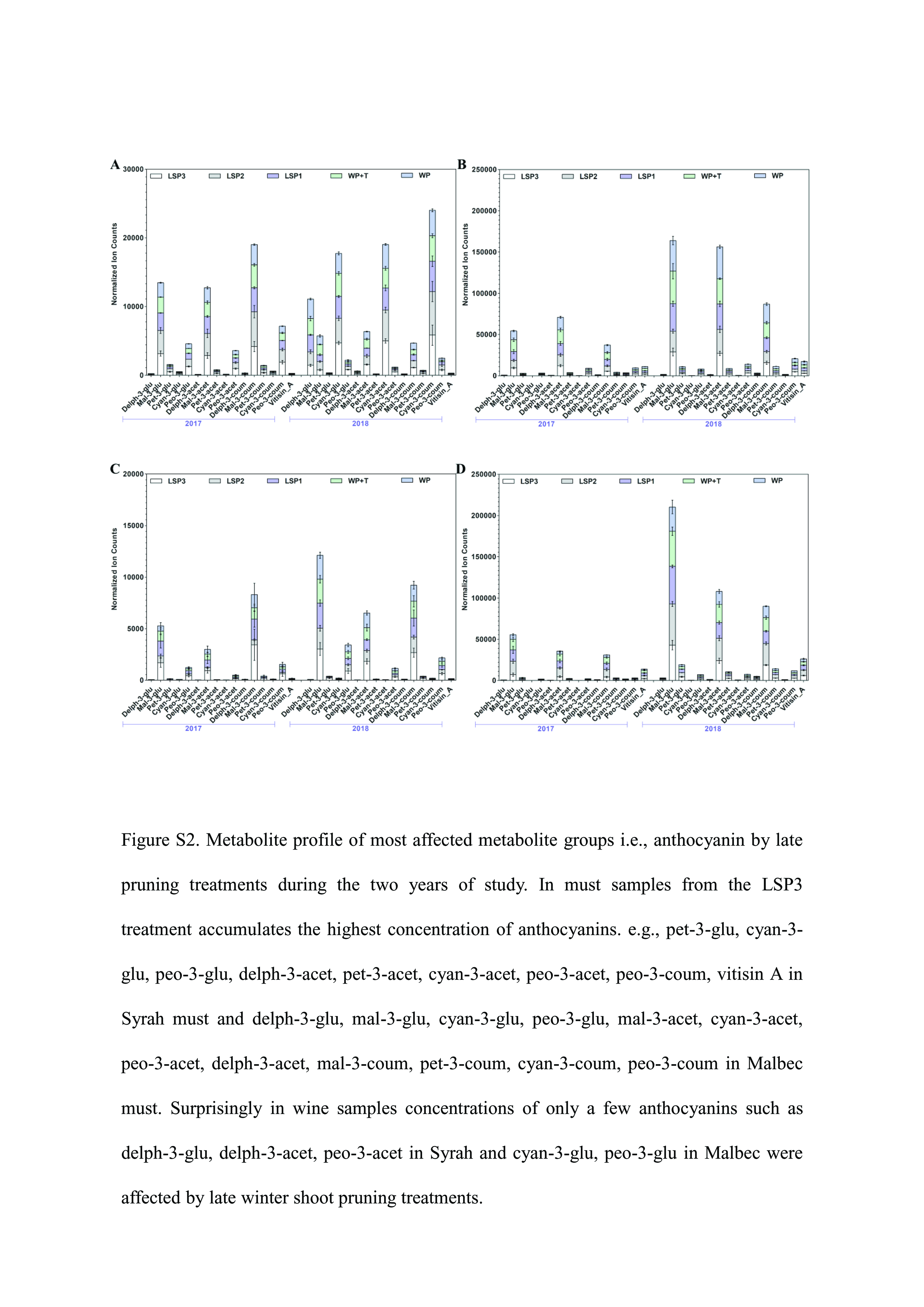

Supplement: Supplementary file 2 [file Image_2.tif]

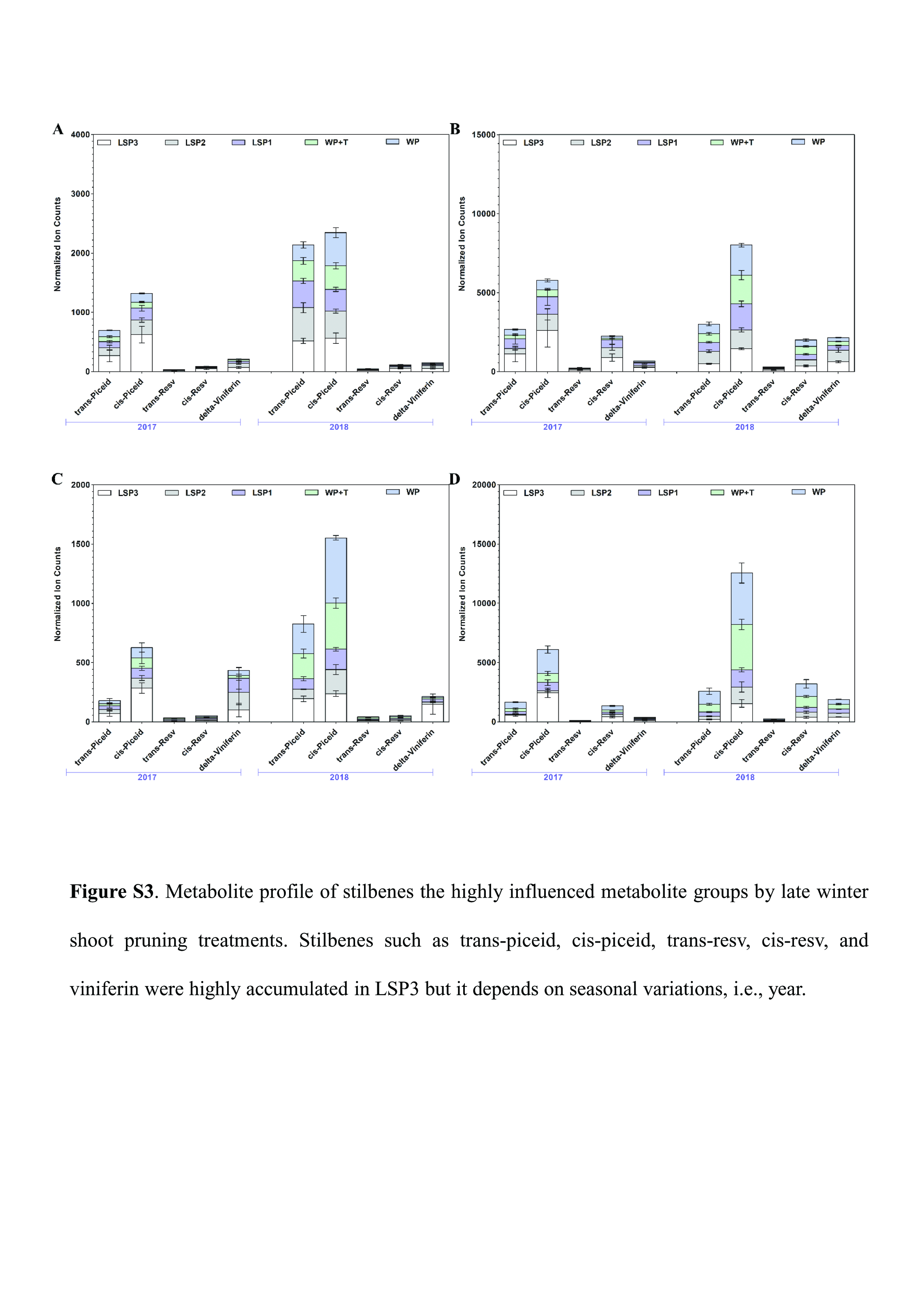

Supplement: Supplementary file 3 [file Image_3.tif]
