# Supplementary material for: Influence of late pruning practice on two red skin grapevine cultivars in a semi-desert climate
Source: Front Plant Sci. 2023 Feb 8;14:1114696. doi: 10.3389/fpls.2023.1114696 (PMC9945113; doi:10.3389/fpls.2023.1114696)
Supplement: Supplementary file 9 [file Table_6.docx]

**Table S6.** UPLC-qTOF-MS method parameters

| **Chromatography** |  |
| --- | --- |
| Liquid chromatography system | Waters Q-TOF Xevo™ |
| Interface | ESI interface |
| Column | Acquity UPLC BEH C18 column (100 mm × 2.1 mm, 1.7 μm) |
| Column temperature | 40 °C |
| Autosampler temperature | 10 °C |
| Injection volume | 4 µL |
| Mobile phase A | 95% water, 5% acetonitrile, 0.1% formic acid |
| Mobile phase B | Acetonitrile containing 0.1% (v/v) formic acid |
| Solvent gradient | 100% - 60% solvent A (8 min)  60% - 0% solvent A (1 min)  100 % solvent A (3.5 min)  100% solvent B (2.5 min, for conditioning) |
| Run time | 15 minutes |
| Flow rate | 0.5 mL/min |
| **Mass Spectrometry** |  |
| Capillary voltage | +3.0 keV |
| Sampling cone voltage | 27 V |
| Extraction cone voltage | 4 V |
| Cone gas flow | 50 Lh^-1^ |
| Desolvation temperature | 300°C |
| Desolvation gas flow | 650 Lh^-1^ |
| Collision energy | 6 eV |
| Source temperature | 120 ◦C |
| MS/MS spectra, collision energies | 25 to 50 eV |
| Scan range | 50 - 1500 m/z |
| Dynamic range enhancement mode | off |
| Lock mass calibration | Leucine enkephalin at a concentration of 0.4 ngl^-1^, in 50/50 acetonitrile/water with 0.1% v/v formic acid |
